# Power asymmetry and embarrassment in shared decision-making: predicting participation preference and decisional conflict

**DOI:** 10.1186/s12911-025-02938-4

**Published:** 2025-03-10

**Authors:** Karin Antonia Scherer, Björn Büdenbender, Anja K. Blum, Britta Grüne, Maximilian C. Kriegmair, Maurice S. Michel, Georg W. Alpers

**Affiliations:** 1 Department of Psychology, School of Social Sciences, University of Mannheim, Mannheim, Germany; 2 Department of Urology and Urosurgery, Medical Faculty Mannheim, University Medical Center Mannheim, Heidelberg University, Mannheim, Germany; 3 Otto Selz Institute, University of Mannheim, Mannheim, Germany

**Keywords:** Shared decision-making, Patient-clinician interaction, Patient participation, Participation preference, Embarrassment, Power asymmetry, Urology, Urooncology

## Abstract

**Background:**

Shared decision-making (SDM) is the gold standard for patient-clinician interaction, yet many patients are not actively involved in medical consultations and hesitate to engage in decisions on their health. Despite considerable efforts to improve implementation, research on barriers to SDM within the patient-clinician relationship and interaction is scant. To identify potential barriers to urological patients’ participation in decision-making, we developed two novel scales assessing power asymmetry (PA-ME) and embarrassment in medical encounters (EmMed). The present study validates both scales in a large sample comprising urological patients and non-clinical participants. It further examines the effects of both factors on participation preferences and decisional conflict among patients.

**Methods:**

Data were collected from 107 urological patients at a university hospital for Urology and Urosurgery in Germany. Patients completed self-report questionnaires before and after their clinical appointments. In addition, 250 non-clinical participants provided data via an online study. All participants rated perceived power asymmetry in the patient-clinician relationship and their experience of embarrassment in medical contexts using the PA-ME and EmMed scales. Urological patients further indicated their participation preference in decisions regarding both general and urological care prior to the consultation. Afterward, they assessed the level of perceived decisional conflict.

**Results:**

Factor analyses yielded power asymmetry and medical embarrassment as unidimensional constructs. Both questionnaires have good (PA-ME; α = 0.88), respectively excellent (EmMed; α = 0.95), internal consistency. Among urological patients, higher levels of perceived power asymmetry predicted lower generic participation preference (β = − 0.98, *p* <.001, adjusted *R*^*2*^ = 0.14) and higher decisional conflict (β = 0.25, *p* <.01, adjusted *R*^*2*^ = 0.07). While, in patients, embarrassment was not linked to generic participation preference before the consultation (*p* ≥.5), it resulted in higher decisional conflict after the consultation (β = 0.39, *p* <.001, adjusted *R*^*2*^ = 0.14). Neither power asymmetry nor embarrassment were specifically associated with participation preference regarding urological care (*p* ≥.273).

**Conclusions:**

Given their promising psychometric properties, the new instruments are recommended for routine assessment of power asymmetry and embarrassment among patients. Addressing these factors may be helpful to reduce decisional conflict and increase participation preferences. Both factors are prerequisites for a successful SDM-process and active patient engagement in health-related decisions.

**Supplementary Information:**

The online version contains supplementary material available at 10.1186/s12911-025-02938-4.

## Background

Shared decision-making (SDM) is the gold standard of patient-clinician interaction [1] and is particularly important for preference-sensitive decisions [2]. In such preference-sensitive decisions, treatment options show comparable efficacy [3, 4], and the benefit-harm ratio depends on patients’ individual priorities and capacities [4, 5]. Consequently, their preferences must be explicitly inquired and considered in the decision-making process [6].

From a practical perspective, SDM positively impacts various patient outcomes, such as knowledge [7, 8], treatment adherence [9–11], and treatment satisfaction [12–14]. Beyond that, it strengthens and respects patients’ right to self-determination and autonomy within medical consultations [1, 15, 16]. Consequentially, SDM has been incorporated into international policy agendas, patient rights, and healthcare education and training [17]. In Germany, SDM is part of the National Cancer Plan, which particularly calls for the practice of SDM in oncological consultations [18].

Moreover, a large proportion of oncological patients prefer SDM in their treatments, with rates ranging from 33% [19] to 46% [20]. Among tumor entities, urooncological patients exhibit particularly high preferences for active participation [20, 21]. Specifically, patients with cancer of the male genital organs demonstrate the highest desire towards autonomy in decision-making [19]. This may be explained by the several preference-sensitive, high-stake decisions urooncological patients face before and during treatment [22, 23]. In the case of early-stage prostate cancer, for example, patients and clinicians must carefully consider the patients’ capacities and preferences when choosing among alternative treatment choices, including active surveillance, surgery, or various forms of radiation treatment [24, 25]. Thus, promoting patients’ autonomy in urological decision-making is crucial, and implementation of SDM is demanded by current urological and urooncological treatment guidelines [26–28]. Consistently, a survey among German urologists indicated that 84% consider SDM the preferred method of patient-clinician communication [29].

However, so far individual patient characteristics or treatment preferences are only partially addressed in oncological care [30]. The percentage of patients who report experiencing SDM ranges from 39% [20] to 51% [19] in oncology populations and around 45% [20, 31] in urooncological settings. Furthermore, despite the significant proportion of cancer patients favoring SDM, about a quarter prefer to not be involved in health decisions [19, 20].

Therefore, a significant step to enhance the implementation of SDM is to identify and address barriers that impede patients’ willingness to engage in health decisions. To date, efforts have primarily focused on the development of clinician guidance and structural requirements, as well as institutional care programs that center on the principles of SDM [17, 32, 33]. However, the inherent dynamics of the patient-clinician relationship and potential impediments within their interactions have been notably overlooked. The patient-clinician relationship is naturally characterized by an imbalance, as patients seek help, expertise, and care that can only be provided by clinicians [34]. However, the perception of power asymmetry and feelings of embarrassment may intensify perceived inferiority and inadequacy among patients, hindering them from expressing preferences, voicing disagreements, and actively participating in the decision-making process. While qualitative patient interviews have yielded initial support for this notion [35], yet to date, there is limited quantitative empirical research on these interactional impediments.

### Power asymmetry as a barrier to SDM

The inherently imbalanced relationship between help-seeking patients and care-providing clinicians [34] has been found to be further reinforced by two critical assumptions held by patients [35]. First, the belief that a “good” patient should be passive and compliant [35]. Often, patients think that active engagement in consultations might be seen as undermining the clinicians’ authority, possibly straining the relationship [36]. Second, patients tend to underestimate their capacity to comprehend medical information provided by the clinician [37] and mistakenly assume that their preferences and personal information are superfluous to the decision-making process [35].

Both assumptions may lead to deferring decisions to the clinician and hinder patients’ engagement in health discussions. Indeed, power asymmetry has been found to prevent patients from speaking up - even when they possess a high level of medical knowledge [36] or have severe concerns about the received quality of care [38–40]. This effect has previously been called *white-coat silence* [41] and is particularly pronounced when the stakes are high, as in the case of severe diseases and heightened dependency on the clinician [1, 42].

Given the life-threatening nature of urooncological conditions, patients in this field likely perceive heightened vulnerability and dependency in their relationship with clinicians. Consequently, this patient population may be especially prone to experiencing power asymmetry, making them particularly relevant for assessing the impact of power asymmetry on engagement in SDM. Indeed, an initial study by Büdenbender et al. [24] found that urological and urooncological patients’ beliefs about their role and acceptable behavior in the interaction and decision-making process were strong predictors of their participation preference in decision‐making. The study used a prior measure of power asymmetry, predominantly examining patients’ positive and negative beliefs about participating in decision-making. This includes thoughts about the advantages of participating, competency beliefs, trust in the clinician, and clinicians’ reactions to participation [43]. However, the scale misses out on patients’ perceived dependency on the clinician, the avoidance of speaking up, and how patients value a good relationship with the clinician. Consequently, to facilitate the assessment of the prevalence and impact of power asymmetry in everyday health practice, we developed the *Power Asymmetry in Medical Encounters (PA-ME)* questionnaire (details on the scale development are provided in the Measures section).

### Embarrassment in medical encounters

In addition to power asymmetry, embarrassment may not only emerge as a highly relevant emotion in the medical context but is also likely to impede patient-clinician communication. As a self-conscious emotion [44], embarrassment typically arises when there is a discrepancy between an individuals’ self-presentation and the perceived societal standard for self-presentation [45]. Distinguished from shame, which is elicited in response to morally wrong or reprehensible actions [46], embarrassment is a response to something that threatens our projected image but is otherwise morally neutral. While both terms are often used interchangeably in the research context of medical encounters, this study specifically concentrates on the nuanced aspects of embarrassment.

Given the intimate and socially sensitive nature of urology, examinations in this specialty are likely to elicit embarrassment in patients. Procedures often involve intimate body areas and require patients to undress, which can elicit substantial embarrassment in patients [47, 48]. Invasive procedures such as urinary catheterization to monitor excretory functions further thread patients’ boundaries and dignity [49]. Beyond that, consultations can encompass intimate or stigmatized topics like the patients’ sexual practices [50], unhealthy lifestyles, or past non-compliance with health advice [49]. Consequently, urological patients appear to be a suitable population to assess the prevalence and impact of embarrassment in medical encounters.

However, empirical research on the occurrence and impact of embarrassment in general medical encounters and urology specifically is rare [51, 52]. Initial studies on patients suffering from urinary incontinence show that noticeable symptoms, like urine odor, wet pants, or frequent daytime urination, induce embarrassment in patients [53, 54]. Crucially, results are limited by their reliance on single-item assessments or qualitative interviews.

Previous measures to quantitatively assess medical embarrassment cover elicitors as the appearance and function of the body, intimate medical examinations, and concerns about judgment or negative evaluation by healthcare providers [55, 56]. However, embarrassment stemming from a lack of knowledge about medical terms, past non-compliance with treatment, or sharing private information has not been addressed. In response to this, we introduce the *Embarrassment in Medical Consultation* (*EmMed*) questionnaire to provide a comprehensive assessment of patients’ experiences with medical embarrassment (details on the scale development are provided in the Measures section). Once validated, the EmMed questionnaire promises to allow for an assessment of medical embarrassment prevalences in urology and facilitates investigations into its impact on SDM. When discussing concerns about medication side effects on sexual functioning, an initial study found embarrassment as a notable impediment to effective communication and information seeking [57]. However, their results were limited by their reliance on qualitative interviews, and the focus on a different medical specialty. Thus, to particularly understand the effect of embarrassment on SDM in urology, further research with a validated psychometric measure is needed.

### Aim of the study and hypotheses

First, we aim to evaluate two novel measures of relevant dynamics within patient-clinician interaction. Specifically, we assess the factor structure and internal consistency of the two newly developed questionnaires on power asymmetry and embarrassment in medical encounters in a large dataset comprising a urological patient sample and a non-clinical sample. In the second step, we investigate how both constructs, power asymmetry and embarrassment, relate to urological patients’ preference towards participation in decisions regarding their general and urological care. As it is a commonly used patient-reported outcome measure of SDM quality [58–60], we further assess both constructs’ relationship to patients’ perceived decisional conflict after consultation.

## Methods

### Participants and procedure

#### Urological sample

Data was collected at the emergency room and the elective outpatient clinic of the Department for Urology and Urosurgery at University Medical Center Mannheim, Germany. Eligible participants were at least 18 years old and fluent in German. A nurse or research assistant approached patients and informed them about the study. After providing informed consent, patients completed a set of self-report questionnaires before and after their appointment with a clinician. While waiting for their appointment (pre-consultation), patients provided sociodemographic data and filled in the German version of the Autonomy Preference Index (API; [61, 62]), the Autonomy Preference Index for Urooncology (API-Uro; [63]) and the newly developed questionnaires on power asymmetry (PA-ME, see Appendix A, Additional File 1) and embarrassment (EmMed, see Appendix B, Additional File 1) in medical encounters. After the consultation, patients were instructed to continue with the German version of the Decisional Conflict Scale (DCS; [64]). Beyond that, clinical information was obtained from the patients’ electronic health records. The study protocol was approved by the ethics committee of the Medical Faculty of Mannheim, University of Heidelberg (amendment to MA-2019–635N). Data collection took place from September 2021 to February 2023. When a study nurse or research assistant was available, patients were invited to participate. This process did not involve any systematic selection beyond the listed eligibility criteria.From an initial recruitment of *n* = 128 urological patients, 21 participants (16.4%) were excluded from analyses: Seven were excluded due to substantial missing data (≥ 60%), three because they had no consultation with a clinician on the day of data acquisition, and another 11 patients due to potentially systematic response patterns. Hence, the final sample consisted of *n* = 107 urological patients. Given the absence of any systematic pattern of missing values in the left-over sample, we used median imputation in cases where a maximum of two items per questionnaire were missing.

The sample was primarily male (*n* = 86 men, 80.4%), of older age (*M* = 61.2, *SD* = 12.1), and of German nationality (*n* = 100, 93.5%). Moreover, 42.1% were oncological patients, and another 18.7% had a tentative cancer diagnosis. Further details on sociodemographic and clinical characteristics, as well as sample comparisons, are reported in Table 1.

**Table 1:** Sociodemographic and clinical characteristics of urological and non-clinical sample and results of sample comparisons

Characteristics	Urological sample *n* = 107		Non-clinical sample *n* = 250		Sample comparison			
	*n*	%	*n*	%	χ²	*df* *(1*,* N =)*	*p*	Cramer’s V*/ OR*
**Gender**								
Female	21	19.6	199	79.6				
Male	86	80.4	48	19.2				
Divers^a^	0	0	3	1.2	115.28	354	< 0.001	0.58/ 6.98
**Nationality**								
German	100	93.5	236	94.4				
Other	7	6.5	14	5.6	0.01	357	0.920	-
**Highest educational level**								
University degree	39	36.5	95	38.0				
No university degree	67	62.6	155	62.0	0.01	356	0.924	-
**Living arrangement**								
Living alone	19	17.8	54	21.6				
Living with others	79	73.8	196	78.4	0.10	348	0.757	-
**Hospital admission**								
Fixed appointment	100	93.5	-	-				
Emergency	2	1.9	-	-				
**Diagnosis**								
Oncological	45	42.1	-	-				
Suspected oncological	20	18.7						
Non-oncological	37	34.3	-	-				
	***M***	***SD***	***M***	***SD***	***t***	***df***	***p***	**Cohen’s** ***d***
Age	61.2	12.1	26.6	9.5	-26.3	164.8	< 0.001	3.34

Note. *n* may vary for cells due to pairwise exclusion and missing values. Welch’s two-sample t-test was conducted to analyze sample differences in age. Pearson’s χ²-tests were conducted to examine sample differences in gender, nationality, educational level, and living arrangement

### Non-clinical sample

To ensure that both scales could be validated in a large and diverse sample, we additionally collected data from a non-clinical sample online via SoSci Survey [65]. The study was advertised on social media, the online research platform SurveyCircle [66], and the local survey system of the University of Mannheim [67]. After giving informed consent, participants provided sociodemographic data and filled in the EmMed and PA-ME questionnaires. In the questionnaires, participants do not need to relate their responses to a current illness or treatment. They are rather asked to express their overall attitudes towards the patient-clinician relationship and involvement in medical decision-making, and their general embarrassment experiences in the medical context, respectively. Thus, we enlarged our sample to validate the novel questionnaires by also administering them to a non-clinical population sample. Data collection was carried out between March 2021 and June 2021.

Of *n* = 265 participants, 15 (5.7%) were excluded due to not passing the attention control item. Hence, the final sample consisted of *n* = 250 subjects. Participants were primarily female (*n* = 199 women, 79.6%), of young age (*M* = 26.6, *SD* = 9.5), and of German nationality (*n* = 236, 94.4%). See Table 1 for further details.

### Measures

#### Power asymmetry in medical encounters (PA-ME)

The PA-ME assesses the patients’ perceived power asymmetry in their relationship with the clinician. Items were developed based on previous research [35, 43, 68] and theoretical considerations regarding elicitors and consequences of perceived power asymmetry. Items were then revised with feedback from urological patients (Selbsthilfe-Bund Blasenkrebs e. V.) [self-help association for bladder cancer]. The examined 20-item version of the questionnaire is reported in Appendix A, Additional File 1. Items assess feelings of social inferiority, patients’ expectations and assumptions about clinicians’ reactions, avoidance behavior caused by perceived power asymmetry, and how important the relationship to and perception by clinicians is to the patients. Items are rated on a 5-point Likert scale from 1 (*strongly disagree*) to 5 (*strongly agree*). Sum scores are calculated and transformed with min-max normalization to range from 0 to 100 (*PA-ME Score*) for comparability to other scores in the research field (e.g. API-Uro; [63]). Higher values indicate higher levels of perceived power asymmetry.

#### Embarrassment in medical consultation (EmMed)

The EmMed assesses perceived embarrassment elicited by lack of medical knowledge, appearance and function of the body, medical examinations, stigmatization of diseases, intimate questions, and missing treatment compliance in the past. Items were developed based on prior research [55, 69] and theoretical considerations regarding the elicitors and effects of embarrassment in medical encounters. After consulting with several psychologists and urologists, items were revised with feedback from urological patients (Selbsthilfe-Bund Blasenkrebs e. V.) [self-help association for bladder cancer]. The examined 21-item version of the questionnaire is reported in Appendix B, Additional File 1. Items are rated on a 5-point Likert scale from 1 (*strongly disagree*) to 5 (*strongly agree*). Sum scores are calculated and transformed with min-max normalization to range from 0 to 100 (*EmMed Score*), with higher values indicating a higher level of perceived embarrassment.

In addition to the EmMed questionnaire, we exploratorily assessed whether the participant has ever shown health-related avoidance behavior (for example, skipped a health appointment or an examination) because of embarrassment provoked by any of the mentioned elicitors (7 items). Items were answered in a dichotomous response format (*yes* vs. *no*). With *no* referring to 0 and *yes* referring to 1, a sum score was built. Ranging from 0 to a maximum of 7, higher scores indicate more avoidance behavior due to embarrassment in the past. Furthermore, we used a single item to exploratorily assess whether the participants’ decision-making was ever impaired by perceived embarrassment. The item is presented with a 5-point Likert response format ranging from 1 (*strongly disagree*) to 5 (*strongly agree*), with higher values indicating more decision impairment. Exploratory results are reported in Appendix C, Additional File 1.

#### Autonomy preference index (API)

We used the German API questionnaire’s decision-making preference subscale (*API-dm*; four inverted items) to assess patients’ generic participation preference [62]. The subscale is commonly used on its own and has been validated in several settings [70, 71]. Responses are made on a Likert scale ranging from 0 (*strongly disagree*) to 4 (*strongly agree*). Sum scores are min-max normalized to range from 0 to 100, with higher values indicating a stronger desire for autonomy. In previous studies, the internal consistency of the German API-dm subscale was high (α = 0.81, [72]; α = 0.85, [62]). This study’s internal consistency was comparable (urological sample: α = 0.82).

#### Autonomy preference index for urooncology (API-Uro)

The API-Uro [63] consists of four vignettes and seven belonging items. Each item represents a putative decision about essential steps in urological or urooncological care ranging from initial diagnosis through critical treatment decisions to follow-up care. Patients indicate who should make the corresponding decision on a 5-point Likert scale ranging from 1 (*physician alone*) to 5 (*patient alone*). Scores of 3 indicate a preference for shared decisions. An additional control item assesses whether patients were able to put themselves in the position of the patient described in the vignettes. A sum score is calculated and transformed with min-max normalization to a range from 0 to 100 for easier interpretation. Higher values indicate a preference for more autonomy. In previous studies, the internal consistency of the scale was satisfying (α = 0.83, [72]; α = 0.92, [63]). In this study’s urological sample, internal consistency was comparable (α = 0.87).

#### Decisional conflict scale (DCS)

We used the German version of the DCS [64] to assess perceived uncertainty about which option to choose and decisional conflict after consultation. The questionnaire consists of 16 items rated on a 5-point Likert scale from 0 (*strongly agree*) to 4 (*strongly disagree*). Sum scores are built, and min-max normalized to range from 0 to 100, with higher values indicating a higher level of decisional conflict and unfavorable decisions. A score ≥ 25 marks a significant decisional conflict associated with decision delay, regret, lower treatment compliance, and a higher intention to sue clinicians in harm cases [73, 74]. The psychometric properties of the DCS have been acceptable in previous samples (average α = 0.88 in a review of 54 studies, [75]). In this study, internal consistency in the urological sample was satisfying (α = 0.96).

### Statistical analyses

We utilized a large dataset collected in the urological sample and the non-clinical sample (*N* = 357) to conduct polychoric exploratory factor analyses (EFAs) to assess the factorial validity of the PA-ME questionnaire and the first subscale of the EmMed questionnaire. Analyses were calculated based on polychoric correlations, which are most appropriate for ordinal-scaled variables [76], and were used for validation of further SDM-related measurements [63]. The eligibility of data for EFA was checked with the Kaiser-Meyer-Olkin measure of sample adequacy (KMO; cut-offs 0.6 = *mediocre*, 0.7 = *middling*, 0.8 = *meritorious*, 0.9 = *marvelous*; [77]) and the Bartlett’s test of sphericity. Subsequently, we followed best practice recommendations [78–80] and calculated the EFAs using the minimal residual (*minres*) extraction method with an oblique rotation (*oblimin*). The optimal number of factors to retain was determined by comparing results of the Scree test, parallel analysis, very simple structure (VSS) criterion, and Velicer’s minimum average partial (MAP) test [79, 80]. The resulting dimensions were built by calculating sum scores. Their reliability is reported in terms of ordinal coefficient α [81, 82].

To investigate the role of power asymmetry and embarrassment as potential barriers to SDM among urological patients, we conducted linear regressions. Specifically, we predicted participation preference regarding decisions in (1) general and (2) urological care and (3) perceived decisional conflict among the urological sample with perceived power asymmetry and embarrassment, respectively. For participation preferences regarding urological care, the sample consisted of patients who stated they could put themselves in the patient’s position described in the vignettes (cutoff: ≥ 4 in control item of the API-Uro). Statistical significance was set at α = 0.05. Where applicable, we report effect sizes [83]. All analyses were conducted in RStudio version 2023.06.2 + 561 [84].

## Results

### Psychometric evaluation of questionnaires

#### Power asymmetry in medical encounters (PA-ME)

Data suitability for conducting the EFA was confirmed with the KMO test of sampling adequacy (KMO = 0.84, *meritorious*; [77]) and a significant Barlett’s test, *χ*²(190) = 2030.93, *p* <.001. As the KMO value of one item was < 0.7, it was removed from analyses [85]. All remaining items had a KMO value greater than 0.7, *range* = [0.71; 0.92]. Thus, no further item was removed.

Results of parallel analysis based on polychoric correlations (for the polychoric correlation matric, see Appendix D, Additional File 1) suggested five factors, but in larger samples parallel analysis likely resembles over-factoring [86]. Results of the Scree test, the VSS criterion, and Velicer’s MAP test allowed for either a one- or a three-factor-solution. As the three-factor-dimensions demonstrated poor internal consistencies and consisted of few items each, we decided to extract one factor, resulting in 28% of explained variance in the PA-ME items. Factor loadings of the items are presented in Table 2. Two further items have been removed due to low factor loadings (≤ 0.3). The standardized ordinal coefficient α of the resulting factor was good (α = 0.88). The final version of the questionnaire will be available in German and English on MADOC (University of Mannheim, https://madoc.bib.uni-mannheim.de/) Research Output Repository.

**Table 2:** Polychoric factor loadings of the PA-ME questionnaire items (in decreasing order of loadings)

Item		Loadings	*h* ^2^	*u* ^2^	*com*
PM_07	I avoid asking the doctors anything out of fear that it could worsen the relationship.	0.81	0.64	0.36	1
PM_18	I avoid disagreeing with doctors even if I have a different opinion.	0.69	0.48	0.52	1
PM_13	I worry that I might be perceived as a bad patient.	0.69	0.48	0.52	1
PM_20	The authority of doctors intimidates me.	0.67	0.45	0.55	1
PM_01	I avoid asking doctors questions because it could undermine their authority.	0.66	0.43	0.57	1
PM_12	Doctors would feel offended if I were to make my own decisions.	0.60	0.36	0.64	1
PM_08^a^	If I had difficulties with a treatment decision, I would express it.	0.59	0.35	0.65	1
PM_15	Doctors would resent me if I were involved in my medical decisions.	0.59	0.35	0.65	1
PM_06	I feel dependent on the goodwill of the doctors when it comes to medical decisions.	0.53	0.28	0.72	1
PM_14	When it comes to medical decisions, I sometimes feel helpless.	0.50	0.25	0.75	1
PM_05	I try not to take up too much time of the medical staff.	0.48	0.23	0.77	1
PM_16^a^	If a treatment was suggested to me, I would dare to ask about other treatment options.	0.46	0.21	0.79	1
PM_17	It is important to me that the doctors like me.	0.45	0.20	0.80	1
PM_09	I always endeavor to cause as few problems as possible for the healthcare staff.	0.41	0.17	0.83	1
PM_03^a^	I have no difficulty in demanding further treatment options from the doctors.	0.41	0.17	0.83	1
PM_02	I don’t feel qualified enough to be involved in medical decisions.	0.35	0.12	0.88	1
PM_11	What the doctors think about me has an influence on the treatment I receive.	0.34	0.12	0.88	1

Note. Polychoric exploratory factor analysis with minimum residual factor extraction and *oblimin* rotation. *N* = 357. Loadings ≤ 0.3 are omitted. Items were presented in German and are translated here

^a^Items inverted before analyses. *h*^*2*^ = communality; *u*^*2*^ = uniqueness; *com* = complexity

#### Embarrassment in medical consultations (EmMed)

Data suitability of the items for calculating the EFA was confirmed by a marvelous KMO measure of sampling adequacy (KMO = 0.93; [77]) and a significant Bartlett’s test, χ²(210) = 3777.51, *p* <.001. All items had a KMO value greater than 0.7, *range* = [0.89; 0.96], thus no item was removed from analyses [85].

Parallel analysis based on polychoric correlations (for the polychoric correlation matric, see Appendix E, Additional File 1) suggested a five-factor structure. However, because in larger samples parallel analysis likely resembles over-factoring [86], we decided to retain one factor in concordance with results of the Scree test, the VSS criterion, and Velicer’s MAP test. The extraction of one factor explained 47% of variance in the EmMed items. Factor loadings are presented in Table 3. The standardized ordinal coefficient α was excellent (α = 0.95). The final version of the questionnaire will be available in German and English on MADOC (University of Mannheim, https://madoc.bib.uni-mannheim.de/) Research Output Repository.

**Table 3:** Polychoric factor loadings of EmMed questionnaire items (in decreasing order of loadings)

Item		Loadings	*h* ^2^	*u* ^2^	*com*
EM01_04	I fear being negatively judged by the doctor when I have to undress for an examination.	0.85	0.71	0.29	1
EM01_05	It makes me uncomfortable to show my body to someone, even if it is a doctor.	0.83	0.69	0.31	1
EM01_10	I feel embarrassed when I am palpated or examined in an intimate area during an examination.	0.83	0.69	0.31	1
EM01_07	I feel ashamed when I have to demonstrate movements or exercises during the examination that are difficult for me.	0.82	0.67	0.33	1
EM01_19	When the doctor asks about my defecation or urination during the appointment, it is embarrassing for me.	0.74	0.54	0.46	1
EM01_06	I feel self-conscious about the appearance of my body.	0.72	0.52	0.48	1
EM01_18	It is very uncomfortable when the doctor asks about my sexuality during the appointment.	0.71	0.51	0.49	1
EM01_13	I feel uncomfortable providing a urine or stool sample for the examination.	0.70	0.50	0.50	1
EM01_11	I feel uncomfortable when examined by a doctor of the opposite gender.	0.70	0.49	0.51	1
EM01_09	I do not want to show any weakness during the appointment.	0.69	0.47	0.53	1
EM01_12	I feel awkward when there is additional staff present during the examination along with the doctor.	0.68	0.46	0.54	1
EM01_08	I feel ridiculous when I have to perform movements or exercises during the examination that are very easy.	0.67	0.45	0.55	1
EM01_03	It is embarrassing when it becomes apparent during the consultation that I misjudged my condition or thought it was worse.	0.66	0.44	0.56	1
EM01_14	I feel ashamed when it is mentioned during the appointment that certain unhealthy behaviors (e.g., smoking, eating) have contributed to my health problems.	0.66	0.43	0.57	1
EM01_01	I feel embarrassed when the doctor asks about my knowledge of my condition, and I cannot answer everything.	0.64	0.40	0.60	1
EM01_17	I feel embarrassed when my condition is associated with physical limitations (e.g., incontinence).	0.61	0.37	0.63	1
EM01_20	I prefer not to be asked personal questions (e.g., about my private life) during the appointment.	0.60	0.36	0.64	1
EM01_21	I feel ashamed when it is addressed during the appointment that I did not follow through with what was agreed upon with the doctor (e.g., medication intake).	0.60	0.36	0.64	1
EM01_15	I would rather not have other people see me in the waiting room.	0.60	0.36	0.64	1
EM01_02	I feel inadequate when the doctor uses complicated medical terms that I don’t understand.	0.47	0.22	0.87	1
EM01_16	When I’m ill, I don’t talk about it.	0.43	0.18	0.82	1

Note. Polychoric exploratory factor analysis with minimum residual factor extraction and *oblimin* rotation. *N* = 357. Loadings ≤ 0.3 are omitted. Items were presented in German and are translated here

*h*^*2*^ = communality; *u*^*2*^ = uniqueness; *com* = complexity

### Prediction of SDM by power asymmetry and embarrassment in urological patients

Descriptive statistics of power asymmetry and embarrassment assessed in both samples are reported in Table 4. In addition, descriptive statistics of the SDM-process variables (API, API-Uro, DCS) assessed in the urological sample are reported. Pearson product-moment correlations of all measures are reported in Appendix F, Additional File 1.

**Table 4:** Descriptive statistics of primary variables in urological and non-clinical sample and results of sample comparisons

	Urological sample *n* = 107		Non-clinical sample *n* = 250		Sample comparisons			
	*M*	*SD*	*M*	*SD*	*t*	*df*	*p*	Cohen’s *d*
PA-ME Score^a^	34.4	11.3	40.4	13.5	4.34	236.9	< 0.001	0.47
EmMed Score^a^	16.6	11.9	37.0	17.8	12.68	290.7	< 0.001	1.26
API^b^	46.6	25.9	-	-	-	-	-	-
API-Uro^c^	38.5	13.3	-	-	-	-	-	-
DCS^d^	12.1	11.3	-	-	-	-	-	-
	**n**	**%**						
significant decisional conflict^e^	23	21.5	-	-				

Note. API, API-Uro, and DCS were assessed in the urological sample only. Diverging cell counts from the sample *n* are due to pairwise exclusion of missing values. Welch’s two-sample t-test was conducted to compare power asymmetry and embarrassment between samples

*M* = Mean. *SD* = Standard Deviation

^a^ Scores min-max-normalized, *range* = [0;100]

^b^ Participation preference. *n* = 105

^c^ Participation preference regarding urological care. *n* = 91

^d^ Decisional conflict. *n* = 94

^e^ Threshold: decisional conflict score ≥ 25

#### Power asymmetry

**Fig. 1:**
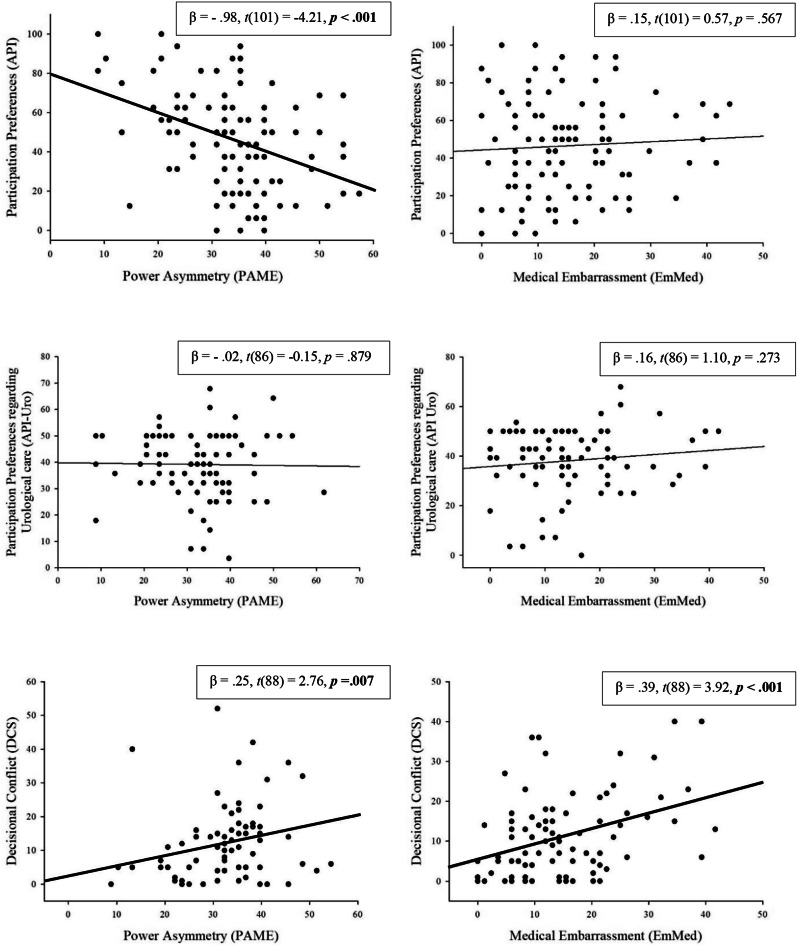
Prediction of SDM variables by power asymmetry and embarrassment in the urological sample Note. Predictions were conducted with linear regression. Bold lines and p-values indicate significant predictions

Higher levels of power asymmetry significantly predicted lower participation preference (API), β = − 0.98, t(101) = -4.21, *p* <.001, and explained a substantial proportion of variance with an adjusted *R*^*2*^ = 0.14, *F* (1,101) = 17.7, *p* <.001 (see Fig. 1). Furthermore, higher levels of power asymmetry significantly predicted greater decisional conflict (DCS), β = 0.25, *t*(88) = 2.76, *p* =.007, and explained a meaningful proportion of variance, adjusted *R*^*2*^ = 0.07, *F* (1,88) = 7.62, *p =*.007 (see Fig. 1). Power asymmetry was no significant predictor for participation preference regarding urological care (API-Uro) (*p* =.825) (see Fig. 1).

#### Embarrassment

Higher levels of embarrassment significantly predicted more decisional conflict (DCS), β = 0.39, *t*(88) = 3.92, *p* <.001, and explained a substantial proportion of variance, with an adjusted *R*^*2*^ = 0.14, *F* (1,88) = 15.36, *p* <.001. However, embarrassment was not a significant predictor for participation preference in general (API, *p* =.567) and regarding urological care (API-Uro, *p* =.273) (see Fig. 1).

## Discussion

SDM is the gold standard of patient-clinician interaction, but several barriers can get in the way. We introduced two short questionnaires to assess power asymmetry and embarrassment in medical encounters. With promising reliability, the questionnaires can be used in routine medical care to assess both constructs in patients. This is crucial given our findings that (a) urological and non-clinical populations perceive power asymmetry and embarrassment in clinical encounters, and (b) both dynamics significantly impact relevant prerequisite and outcome variables of the SDM process. We found that patients with limiting assumptions about power asymmetry are less inclined to engage in SDM and experience more decisional conflict following consultation with their clinician. Moreover, those who report being embarrassed will later experience more decisional conflict after consultation.

The PA-ME questionnaire demonstrated promising internal consistency, surpassing previous measures (standardized ordinal coefficient α = 0.88 vs. Cronbach’s α = 0.72 in [43]). By incorporating items on additional aspects, the introduced questionnaire comprehensively evaluates perceived power asymmetry, addressing facets untouched by earlier tools [43]. Exploratory analyses suggested the presence of additional dimensions in the PA-ME questionnaires. However, they exhibited poor internal consistencies and were characterized by a reduced number of items. Consequently, we decided to model the questionnaire as a unidimensional measurement of power asymmetry. Further research is crucial to fully understand and differentially explore the facets our research hinted at. As our sample was highly heterogeneous and included urological and non-clinical participants, future analyses with separate, more homogeneous samples will enhance our understanding of power asymmetry’s factor structure, ensuring the questionnaire’s validity across diverse populations.

Similarly, the EmMed questionnaire exhibits excellent internal consistency and covers elicitors of embarrassment not covered by previous measures to assess patient embarrassment [55]. We did not identify distinct dimensions of medical embarrassment, as previously observed in other assessments [55]. Confirmatory studies with homogenous study populations and further exploratory studies including the questionnaire sections on related avoidance behavior and decision-making impairment will be valuable in further exploring the structure and effects of medical embarrassment.

Finally, the PA-ME and EmMed questionnaires facilitate the routine assessment of power asymmetry and embarrassment within patients, enabling its effective addressing during consultations. This is highly recommended, given our findings on their substantial impact on the SDM process and decision-making outcomes.

### Power asymmetry as a barrier to SDM in urological patients

In our patient sample, mean scores of both participation preference scales indicate that patients preferred SDM as decision-making model in general and urological care [61, 62]. However, perceived power asymmetry predicted a decrease in generic participation preference among patients. This highlights the profound impact even subtle power imbalances might have and emphasizes the relevance of this variable to the implementation of SDM in medical consultations. This is also supported by the alignment of our findings with initial research on the effects of power asymmetry on patients’ autonomy preferences [24, 35, 87].

Moreover, our data yielded an effect of power asymmetry on decisional conflict among patients after their consultation with a clinician. This suggests that power imbalances not only decrease patients’ participation preferences but might discourage patients from engaging in decision-making during their appointments. This, in turn, may result in decisions that are less aligned with patients’ values and preferences, as indicated by our findings. The majority of patients in our study reported moderate levels of decisional conflict (*M*_*uro*_ = 12.06, *SD* = 11.28), which is in line with previous findings (*N* = 313, *M* = 18.02, *SD* = 17.47; [59]). Still, 21.5% of the patients we surveyed scored at or above the threshold for significant decisional conflict, which is linked to delaying decisions and experiencing regret [72]. This further underlines the need to address barriers that predict patient decisional conflict. Importantly, while we assessed participation preferences before the consultation and decisional conflict as an outcome of decision-making after the consultation, we did not evaluate the actual occurrence of SDM during the consultation. As this work has identified that embarrassment and power asymmetry influence the outcome of decision-making, future research should address the mechanisms of power asymmetry during the actual consultation with the physician.

We did not find a significant prediction of participation preference regarding urological care (API-Uro) through power asymmetry. Urological patients may have specific concerns about their treatment options before consultation and feel a greater need to address them. Given the significant impact of urological, and particularly urooncological, conditions on patients’ lives [25] patients may feel a greater awareness of the importance of engaging in decision-making. This, in turn, may result in a less inhibited preference to participate in urological treatment decisions, in contrast to the need to participate in decisions in general.

### Embarrassment as a barrier to SDM in urological patients

Our findings suggest that patients who experience embarrassment in medical encounters will later report more decisional conflict after consultation with their clinicians. However, interestingly embarrassment did not impact patients’ preferences for participating in decisions regarding general or urological care, which aligns with prior findings using the same outcome measure [88]. However, Hamann et al. [88] found that embarrassment indeed links to less critical and participatory communication among patients. As these are crucial prerequires, this may ultimately hinder SDM during consultations. This converges with our finding that embarrassment among urological patients predicts greater decisional conflict after counseling: Without ruling out their participation preference, embarrassment may impede patients’ ability to express their preferences effectively, leading to decisions that do not align with patients’ priorities and increased decisional conflict. High or moderate levels of participation preferences do not necessarily mean more participation, and a mismatch between preferred and actual involvement is not uncommon [19]. Our exploratory finding on moderate scores of decision-making impairments due to embarrassment in both samples (see Appendix C, Additional File 1) further supports this hypothesis. Future research should consider and test these assumptions. To gain deeper insights, future research should also incorporate measurements of SDM occurrence and patient participation during the consultation.

Power asymmetry and embarrassment are both dynamics that come into play in the direct interaction and communication between patients and clinicians and can be regarded as predominantly interpersonal barriers to SDM. Also, both barriers contribute to patients’ feelings of insufficiency and inferiority. While the primary focus of this study was not to investigate their specific relationship, we discovered a significant positive correlation between both constructs, *r*(105) = 0.56, *p* <.001 (see Appendix F, Additional File 1), suggesting that both barriers are closely related. It is thus plausible that power asymmetries also affect embarrassments’ impact on communication. When patients initially feel embarrassed, their willingness to engage in SDM may increase if the clinician is attentive to their concerns and open to involving them in the decision. However, embarrassed patients who interact more passively are likely to have less influence on their treatment, which can perpetuate power imbalances. Conversely, interventions that address power asymmetry, embarrassment, or patient empowerment in general may have mutually reinforcing effects, resulting in improved overall health outcomes [89] and well-being [23].

### Limitations and outlook

We assessed the impact of power asymmetry and embarrassment in a urological and urooncological sample, as we expected this patient group to be especially prone to experiencing both barriers. Our patient sample was representative of urological and urooncological patients, being primarily male and aged over 60 [90]. However, to generalize our findings to other patient groups, the questionnaires should be validated in further patient samples.

To validate our questionnaires, we calculated the EFAs with a large and diverse dataset comprising patient and non-clinical participants. This allowed for increasing data variability and higher correlation coefficients [91]. Yet, there may be differences in factor structures between patient and non-clinical samples. The limited sample size of the urological sample did not allow separate analyses. However, exploratory analyses with the non-clinical sample only revealed comparable suggestions for factorial structures (see Appendix G, Additional File 1). Nevertheless, sample-specific factorial structures of both questionnaires should be further examined in future studies. The limited sample size of urological patients may also have hindered us from detecting smaller effects due to a lack of statistical power. Future studies with larger sample sizes may uncover further links between both barriers and the SDM process, particularly regarding the relationship between embarrassment and participation preferences.

We found varying levels of power asymmetry and embarrassment among patients and non-clinical participants. However, samples differed significantly regarding sociodemographic characteristics. Also, while patients sought medical advice for serious urological and oncological conditions, non-clinical participants did not currently face health threats. Thus, although our questionnaires evaluate general attitudes and experiences, caution is needed in interpreting sample differences. Nevertheless, patients may possess extensive knowledge regarding their condition and treatment options, greater familiarity with medical procedures, and a better understanding of what to expect in medical encounters than non-clinical individuals. This may contribute to patients feeling more prepared for health discussions and decisions with their clinicians. Moreover, patients with a lengthy medical history may have established relationships and trust with healthcare providers. This can facilitate discussions on personal issues and foster a balanced power dynamic during consultations. Our data did not allow for an analysis of how the relationship duration or the number of previous appointments influenced the level of perceived embarrassment and power asymmetry. Thus, these considerations require further research. This is particularly interesting because familiarity and trust offer potential targets for interventions to reduce interpersonal barriers to SDM.

Our results are based on self-report data, a common method to assess patient participation. However, self-report data may be biased by social desirability. Although participants were informed that their answers would not be accessible to their clinician, it is plausible that they were still hesitant to report embarrassment, power asymmetry, or decisional conflict in or after the consultation with their clinician. This might help explain the comparably low levels of these measures in the patient sample.

When measuring patients’ participation preferences before consultations in which high-stake decisions are made, patients may not yet be fully aware of their options and are forced to estimate their preferences [92]. Also, when completing questionnaires before the consultation, patients may be in a less emotionally aroused state (*cold*) compared to the actual discussion about their healthcare journey (*hot*). This may create a disparity between patients’ hypothetical and actual preferences, referred to as the *hot-to-cold empathy gap* [70, 93]. However, the case vignettes in the API-Uro questionnaire represent common decisions in urological care [63], which might have reduced biases in the reported participation preferences. Also, we assessed participation preferences shortly before the decision-making situation to minimize the impact of an empathy gap on the general participation preference measure (API).

In our regression models predicting participation preference and decisional conflict, power asymmetry and embarrassment explained only a small proportion of variance. However, participation preference and decisional conflict are multifaceted phenomena, and one cannot expect a single factor to explain large amounts of variance. Previous studies examining predictors of participation preferences and decisional conflict have found comparable proportions of explained variance (participation preference: adjusted *R*^*2*^ = 0.03 − 0.25; [94–96]; decisional conflict: adjusted *R*^*2*^ = 0.1 − 0.12; [59]).

### Practical implications

SDM fosters choices that align with the values and unique needs of patients. However, simply introducing SDM in consultations may be ineffective. SDM consultations differ considerably from the appointments many patients are used to, and a substantial part of patients might have no SDM experience [97]. Thus, patients need to be prepared, with particular caution to the impact of power asymmetry and embarrassment on patients’ capability to engage in decisions.

Current interventions in general healthcare [87] and urology [31] do not address power asymmetries in the patient-clinician-relationship. Patients’ perceptions of their role as inferior in the decision-making process must be challenged, and their concept of *good* (passive and compliant) patients must be redefined. Explicit encouragement is an effective facilitator of involvement in SDM [98, 99]. Thus, patients should explicitly be encouraged to step out of their passive role and engage in critical conversations [35]. Clinicians reassuring patients that active participation is valuable and will not lead to negative outcomes can be a simple yet effective intervention. Online self-help groups can further reduce negative attitudes [100]. Pre-consultation interventions, like coaching sessions and patient activation [101, 102] to foster patients’ self-efficacy and confidence, have been embedded, but their integration into routine healthcare is challenging due to time constraints [35]. Therefore, alternative methods should be investigated. As power dynamics involve both patient and clinician, interventions should also target clinicians. Providers can use the PA-ME questionnaire to assess power asymmetry as perceived by their patients. This may raise awareness and encourage clinicians to reduce power asymmetry in their consultations.

Patients who experience heightened levels of embarrassment require exceptional support and active involvement. A strong patient-clinician relationship, coupled with effective communication skills of the clinician, can help patients overcome embarrassment when discussing intimate topics [103]. Thus, providers should invest time in building a trusting relationship with patients. Active listening, empathy, and respect are essential to facilitate open communication. Clinicians can also openly acknowledge that embarrassment in medical settings is common, thereby normalizing patients’ experiences. Training programs to enhance communication skills, build trustworthy relationships, and create supportive environments should be implemented into clinicians’ curricula. Healthcare services can educate patients about what to expect during medical encounters, including the procedures involved and what will be expected of them. Institutional approaches that incorporate SDM principles into oncological care may also help alleviate fears and concerns.

## Conclusions

Our findings confirm that power asymmetry and embarrassment in the medical setting are relevant aspects in patient-clinician interactions. We provide instruments to reliably measure the constructs in future research and in routine care. To enable patients to actively participate in important medical decisions with potentially life-altering consequences, it’s crucial to address both interpersonal dynamics through practical interventions. Ultimately, this may help ensure patients’ autonomy and self-determination and lead to improved health outcomes and patient satisfaction.

## Electronic supplementary material

Below is the link to the electronic supplementary material.

Supplementary Material 1

## Data Availability

The data that support the findings of this study will be deposited on MADATA (University of Mannheim, https://madata.bib.uni-mannheim.de/) Research Data Repository and made available by the authors, without undue reservation, to any qualified researcher. The final versions of the EmMed and the PA-ME questionnaire will be available in German and English on MADOC (University of Mannheim, https://madoc.bib.uni-mannheim.de/) Research Output Repository.

## Abbreviations

SDM: Shared decision–making
PA-ME: Power Asymmetry in Medical Encounters
EmMed: Embarrassment in Medical Consultation
API: Autonomy Preference Index
API-Uro: Autonomy Preference Index for Urooncology
DCS: Decisional Conflict Scale
EFA: Exploratory factor analysis
KMO: Kaiser–Meyer–Olkin measure of sample adequacy
VSS criterion: Very simple structure criterion
MAP test: Velicer’s minimum average partial test
