# Supplementary material for: Power asymmetry and embarrassment in shared decision-making: predicting participation preference and decisional conflict
Source: BMC Med Inform Decis Mak. 2025 Mar 10;25:120. doi: 10.1186/s12911-025-02938-4 (PMC11892210; doi:10.1186/s12911-025-02938-4)
Supplement: Supplementary file 1 — Supplementary Material 1 [file 12911_2025_2938_MOESM1_ESM.docx]

**Additional file 1**

**Appendix A –** **Examined version of the PAME questionnaire including univariate descriptive statistics in urological and non-clinical sample**

The final version of the questionnaire will be available in German and English on MADOC (University of Mannheim, https://madoc.bib.uni-mannheim.de/) Research Output Repository.

**Supplementary Table 1**

*Examined version of the PAME questionnaire including univariate descriptive statistics in urological and non-clinical sample*

|  |  | *Urological sample* | | *Non-clinical sample* | |
| --- | --- | --- | --- | --- | --- |
| Item |  | *M* | *SD* | *M* | *SD* |
| **PM_01** | I avoid asking doctors questions because it could undermine their authority. | 1.56 | 0.84 | 1.70 | 0.90 |
| **PM_02** | I don't feel qualified enough to be involved in medical decisions. | 2.48 | 1.13 | 2.58 | 1.04 |
| **PM_03^a^** | I have no difficulty in demanding further treatment options from the doctors. | 2.22 | 1.11 | 2.48 | 1.02 |
| PM_04 | I believe the relationship with the doctors has an impact on my treatment. | 3.76 | 1.02 | 3.88 | 0.98 |
| **PM_05** | I try not to take up too much time of the medical staff. | 3.07 | 0.91 | 3.26 | 1.07 |
| **PM_06** | I feel dependent on the goodwill of the doctors when it comes to medical decisions. | 2.71 | 1.06 | 3.18 | 1.09 |
| **PM_07** | I avoid asking the doctors anything out of fear that it could worsen the relationship. | 1.64 | 0.79 | 1.91 | 1.00 |
| **PM_08^a^** | If I had difficulties with a treatment decision, I would express it. | 1.67 | 0.77 | 1.89 | 0.71 |
| **PM_09** | I always endeavor to cause as few problems as possible for the healthcare staff. | 3.55 | 1.03 | 3.58 | 1.01 |
| PM_10 | The opinion of the doctors about me is very important to me. | 3.86 | 0.88 | 3.20 | 1.01 |
| **PM_11** | What the doctors think about me has an influence on the treatment I receive. | 3.11 | 0.99 | 3.19 | 1.08 |
| **PM_12** | Doctors would feel offended if I were to make my own decisions. | 2.47 | 0.97 | 2.79 | 1.05 |
| **PM_13** | I worry that I might be perceived as a bad patient. | 2.12 | 0.98 | 2.39 | 1.13 |
| **PM_14** | When it comes to medical decisions, I sometimes feel helpless. | 2.73 | 0.93 | 3.30 | 1.08 |
| **PM_15** | Doctors would resent me if I were involved in my medical decisions. | 2.03 | 0.82 | 2.16 | 0.94 |
| **PM_16^a^** | If a treatment was suggested to me, I would dare to ask about other treatment options. | 1.82 | 0.83 | 1.98 | 0.83 |
| **PM_17** | It is important to me that the doctors like me. | 3.15 | 0.86 | 3.15 | 0.92 |
| **PM_18** | I avoid disagreeing with doctors even if I have a different opinion. | 2.22 | 0.87 | 2.42 | 0.93 |
| PM_19 | I always strive to have a good relationship with the medical staff. | 3.96 | 0.76 | 4.06 | 0.68 |
| **PM_20** | The authority of doctors intimidates me. | 1.84 | 0.78 | 2.53 | 1.08 |

*Note.* Items presented in the same order as in questionnaire. Urological sample: n = 107. Non-clinical sample: n = 250.

*M* = Mean. *SD* = Standard Deviation. **Bold** **items** are part of the final questionnaire. Non-bold items were excluded from final questionnaire due to KMO value ≤ .7 or factor loadings < .3. ^a^Items inverted before analyses.

**Appendix B - Examined version of the EmMed questionnaire including univariate descriptive statistics in urological and non-clinical sample**

The final version of the questionnaire will be available in German and English on MADOC (University of Mannheim, https://madoc.bib.uni-mannheim.de/) Research Output Repository.

**Supplementary Table 2**

*Examined version of the EmMed questionnaire including univariate descriptive statistics in urological and non-clinical sample*

|  |  | *Urological sample* | | *Non-clinical sample* | |
| --- | --- | --- | --- | --- | --- |
| Item |  | *M* | *SD* | *M* | *SD* |
| **EM01_01** | I feel embarrassed when the doctor asks about my knowledge of my condition, and I cannot answer everything. | 1.79 | 0.95 | 2,67 | 1,19 |
| **EM01_02** | I feel inadequate when the doctor uses complicated medical terms that I don't understand. | 2.42 | 1.16 | 2,78 | 1,17 |
| **EM01_03** | It is embarrassing when it becomes apparent during the consultation that I misjudged my condition or thought it was worse. | 1.79 | 0.83 | 2,68 | 1,15 |
| **EM01_04** | I fear being negatively judged by the doctor when I have to undress for an examination. | 1.32 | 0.62 | 2,12 | 1,15 |
| **EM01_05** | It makes me uncomfortable to show my body to someone, even if it is a doctor. | 1.43 | 0.77 | 2,66 | 1,31 |
| **EM01_06** | I feel self-conscious about the appearance of my body. | 1.44 | 0.79 | 2,07 | 1,19 |
| **EM01_07** | I feel ashamed when I have to demonstrate movements or exercises during the examination that are difficult for me. | 1.43 | 0.67 | 2,32 | 1,14 |
| **EM01_08** | I feel ridiculous when I have to perform movements or exercises during the examination that are very easy. | 1.38 | 0.72 | 1,98 | 1,01 |
| **EM01_09** | I do not want to show any weakness during the appointment. | 1.64 | 0.77 | 2,28 | 1,08 |
| **EM01_10** | I feel embarrassed when I am palpated or examined in an intimate area during an examination. | 1.69 | 0.87 | 2,94 | 1,34 |
| **EM01_11** | I feel uncomfortable when examined by a doctor of the opposite gender. | 1.68 | 0.86 | 2,64 | 1,30 |
| **EM01_12** | I feel awkward when there is additional staff present during the examination along with the doctor. | 1.84 | 0.89 | 2,84 | 1,22 |
| **EM01_13** | I feel uncomfortable providing a urine or stool sample for the examination. | 1.32 | 0.67 | 2,55 | 1,28 |
| **EM01_14** | I feel ashamed when it is mentioned during the appointment that certain unhealthy behaviors (e.g., smoking, eating) have contributed to my health problems. | 1.66 | 0.87 | 2,72 | 1,17 |
| **EM01_15** | I would rather not have other people see me in the waiting room. | 1.30 | 0.57 | 2,18 | 1,26 |
| **EM01_16** | When I'm ill, I don't talk about it. | 2.00 | 0.93 | 2,12 | 1,00 |
| **EM01_17** | I feel embarrassed when my condition is associated with physical limitations (e.g., incontinence). | 2.33 | 1.02 | 2,98 | 1,20 |
| **EM01_18** | It is very uncomfortable when the doctor asks about my sexuality during the appointment. | 1.59 | 0.82 | 2,39 | 1,24 |
| **EM01_19** | When the doctor asks about my defecation or urination during the appointment, it is embarrassing for me. | 1.27 | 0.61 | 2,09 | 1,02 |
| **EM01_20** | I prefer not to be asked personal questions (e.g., about my private life) during the appointment. | 1.54 | 0.76 | 2,04 | 1,08 |
| **EM01_21** | I feel ashamed when it is addressed during the appointment that I did not follow through with what was agreed upon with the doctor (e.g., medication intake). | 2.06 | 1.07 | 3,04 | 1,12 |

*Note.* Items presented in the same order as in questionnaire. Urological sample: n = 107. Non-clinical sample: n = 250.

*M* = Mean. *SD* = Standard Deviation. **Bold** **items** are part of the final questionnaire.

**Appendix C - Results of exploratory analyses on embarrassment-related avoidance behavior and decision-making impairment in both samples**

Analyses on embarrassment-related avoidance behavior and decision-making impairment consist of few exploratory items only that have not been psychometricly validated. Thus, results must be considered with caution. However, we report exploratory findings on the prevalences of both among urological patients and non-clinical participants.

**Supplementary Table 3**

*Exploratory items on avoidance behavior including univariate descriptive statistics in urological and non-clinical sample*

| Due to embarrassment or feelings of shame, have you ever in the past... | | *Urological sample* | | *Non-clinical sample* | |
| --- | --- | --- | --- | --- | --- |
| Item |  | *M* | *SD* | *M* | *SD* |
| EM02_01 | ...missed a necessary doctor's appointment? | 1.05 | 0.21 | 1,21 | 0,41 |
| EM02_02 | ...declined an examination during a doctor's visit? | 1.01 | 0.10 | 1,10 | 0,30 |
| EM02_03 | ...not fully reported symptoms or complaints to your doctor? | 1.06 | 0.23 | 1,30 | 0,46 |
| EM02_04 | ...downplayed symptoms or complaints to your doctor? | 1.11 | 0.32 | 1,34 | 0,47 |
| EM02_05 | ...not asked for clarification when you didn’t really understand something important? | 1.17 | 0.38 | 1,49 | 0,50 |
| EM02_06 | ...not answered certain questions from your doctor? | 1.03 | 0.17 | 1,06 | 0,24 |
| EM02_07 | ...not informed your doctor that you did not adhere to certain measures (e.g., medication intake)? | 1.10 | 0.31 | 1,28 | 0,45 |

*Note.* Items presented in the same order as in study. Urological sample: n = 107. Non-clinical sample: n = 250.

*M* = Mean. *SD* = Standard Deviation.

**Supplementary Table 4**

*Exploratory item on decision-making impairment including univariate descriptive statistics in urological and non-clinical sample*

| Due to the behaviors described above, have you ever had the impression in the past... | | *Urological sample* | | *Non-clinical sample* | |
| --- | --- | --- | --- | --- | --- |
| Item |  | *M* | *SD* | *M* | *SD* |
| EM03_01 | …that the decision-making process regarding further treatment/therapy with your doctor was impaired? | 1.54 | 0.77 | 1,96 | 0,89 |

*Note.* Urological sample: n = 107. Non-clinical sample: n = 250.

*M* = Mean. *SD* = Standard Deviation.

**Supplementary Table 5**

*Sum scores of avoidance-behavior and decision-making impairment in urological and non-clinical sample and sample comparisons*

|  | *Urological sample*  *n = 107* | | | | *Non-clinical sample*  *n = 250* | | | | | | *Sample comparisons* | | | | | | |
| --- | --- | --- | --- | --- | --- | --- | --- | --- | --- | --- | --- | --- | --- | --- | --- | --- | --- |
| Variable | *n* | *M* | | *SD* | *n* | | *M* | | | *SD* | *t* | *df* | | *p* | | *d* | |
| avoidance behavior^a^ | 107 | 0.52 | | 1.15 | 249 | | 1.76 | | | 1.61 | 8.22 | 274.87 | | < .001 | | 0.84 | |
| decision-making impairment^b^ | 107 | 1.54 | | 0.77 | 246 | | 1.95 | | | 0.88 | 4.15 | 351 | | < .001 | | 0.48 | |
|  |  | |  | | |  | |  |  | | | |  | |  | |  |

*Note.* Diverging cell counts from the total sample *n* are due to pairwise exclusion of missing values. Welch’s two-sample t-test was conducted to compare avoidance behavior between samples. Student’s t-Test was conducted to compare decision impairment between samples.

*M* = Mean. *SD* = Standard Deviation. *^a^range* = [1;7]. *^b^range* = [1;5].

**Appendix D - Polychoric correlation matrix of PA-ME questionnaire items**

**Supplementary Table 6**

*Polychoric correlation matrix of PA-ME questionnaire items*

| Item | **PM_01** | **PM_02** | **PM_03^a^** | PM_04 | **PM_05** | **PM_06** | **PM_07** | **PM_08^a^** | **PM_09** | PM_10 | **PM_11** | **PM_12** | **PM_13** | **PM_14** | **PM_15** | **PM_16^a^** | **PM_17** | **PM_18** | PM_19 | **PM_20** |
| --- | --- | --- | --- | --- | --- | --- | --- | --- | --- | --- | --- | --- | --- | --- | --- | --- | --- | --- | --- | --- |
| **PM_01** | 1.00* |  |  |  |  |  |  |  |  |  |  |  |  |  |  |  |  |  |  |  |
| **PM_02** | 0.40* | 1.00* |  |  |  |  |  |  |  |  |  |  |  |  |  |  |  |  |  |  |
| **PM_03^a^** | 0.28* | 0.16* | 1.00* |  |  |  |  |  |  |  |  |  |  |  |  |  |  |  |  |  |
| PM_04 | 0.03 | -0.06 | -0.25* | 1.00* |  |  |  |  |  |  |  |  |  |  |  |  |  |  |  |  |
| **PM_05** | 0.32* | 0.14* | 0.23* | 0.08 | 1.00* |  |  |  |  |  |  |  |  |  |  |  |  |  |  |  |
| **PM_06** | 0.29* | 0.08 | 0.05 | 0.39* | 0.20* | 1.00* |  |  |  |  |  |  |  |  |  |  |  |  |  |  |
| **PM_07** | 0.65* | 0.24* | 0.37* | 0.12* | 0.39* | 0.42* | 1.00* |  |  |  |  |  |  |  |  |  |  |  |  |  |
| **PM_08^a^** | 0.43* | 0.20* | 0.45* | 0.00 | 0.37* | 0.22* | 0.54* | 1.00* |  |  |  |  |  |  |  |  |  |  |  |  |
| **PM_09** | 0.26* | 0.16* | 0.19* | 0.06 | 0.61* | 0.15* | 0.33* | 0.16* | 1.00* |  |  |  |  |  |  |  |  |  |  |  |
| PM_10 | 0.15* | 0.11* | 0.08 | 0.15* | 0.08 | 0.14* | 0.22* | 0.00 | 0.24* | 1.00* |  |  |  |  |  |  |  |  |  |  |
| **PM_11** | 0.15* | -0.09* | -0.02 | 0.54* | 0.06 | 0.44* | 0.25* | 0.12* | 0.10* | 0.23* | 1.00* |  |  |  |  |  |  |  |  |  |
| **PM_12** | 0.34* | 0.15* | 0.10 | 0.16* | 0.19* | 0.50* | 0.42* | 0.29* | 0.17 | 0.12* | 0.39* | 1.00* |  |  |  |  |  |  |  |  |
| **PM_13** | 0.40* | 0.21* | 0.25* | 0.10 | 0.38* | 0.37* | 0.53* | 0.35* | 0.27* | 0.26* | 0.35* | 0.48* | 1.00* |  |  |  |  |  |  |  |
| **PM_14** | 0.29* | 0.29* | 0.18* | 0.15* | 0.17* | 0.38* | 0.34* | 0.28* | 0.17* | 0.05 | 0.14* | 0.27* | 0.35* | 1.00* |  |  |  |  |  |  |
| **PM_15** | 0.39* | 0.21* | 0.15* | 0.04 | 0.23* | 0.40* | 0.46* | 0.32* | 0.08 | 0.12* | 0.29* | 0.66* | 0.44* | 0.26* | 1.00* |  |  |  |  |  |
| **PM_16^a^** | 0.34* | 0.24* | 0.45* | -0.08 | 0.21* | 0.12* | 0.40* | 0.49* | 0.15* | 0.04 | -0.06 | 0.19* | 0.24* | 0.13* | 0.27* | 1.00* |  |  |  |  |
| **PM_17** | 0.20* | 0.04 | 0.03 | 0.30* | 0.26* | 0.30* | 0.30* | 0.13* | 0.35* | 0.50* | 0.36* | 0.28* | 0.48* | 0.21* | 0.17* | 0.09 | 1.00* |  |  |  |
| **PM_18** | 0.44* | 0.30* | 0.40* | -0.02 | 0.29* | 0.30* | 0.55* | 0.45* | 0.29* | 0.24* | 0.15* | 0.41* | 0.41* | 0.40* | 0.36* | 0.44* | 0.25* | 1.00* |  |  |
| PM_19 | 0.04 | -0.07 | -0.04 | 0.25* | 0.25* | 0.11* | 0.07 | 0.02 | 0.41* | 0.19* | 0.22* | 0.09 | 0.22* | 0.10 | -0.05 | -0.15* | 0.39* | 0.01 | 1.00* |  |
| **PM_20** | 0.46* | 0.30* | 0.29* | 0.08 | 0.23* | 0.40* | 0.54* | 0.41* | 0.16* | 0.06 | 0.18* | 0.38* | 0.42* | 0.54* | 0.38* | 0.36* | 0.24* | 0.51* | -0.02 | 1.00* |

*Note.* *N* = 357. Items presented in the same order as in the questionnaire.

**Bold** **items** are part of the final questionnaire. ^a^Items inverted before analyses. *Polychoric correlations significant at level p < .05.

**Appendix E - Polychoric correlation matrix of EmMed questionnaire items**

**Supplementary Table 7**

*Polychoric correlation matrix of EmMed questionnaire items*

| Item: EM01_ | **01** | **02** | **03** | **04** | **05** | **06** | **07** | **08** | **09** | **10** | **11** | **12** | **13** | **14** | **15** | **16** | **17** | **18** | **19** | **20** | **21** |
| --- | --- | --- | --- | --- | --- | --- | --- | --- | --- | --- | --- | --- | --- | --- | --- | --- | --- | --- | --- | --- | --- |
| **01** | 1,00 |  |  |  |  |  |  |  |  |  |  |  |  |  |  |  |  |  |  |  |  |
| **02** | 0,56 | 1,00 |  |  |  |  |  |  |  |  |  |  |  |  |  |  |  |  |  |  |  |
| **03** | 0,63 | 0,51 | 1,00 |  |  |  |  |  |  |  |  |  |  |  |  |  |  |  |  |  |  |
| **04** | 0,55 | 0,40 | 0,56 | 1,00 |  |  |  |  |  |  |  |  |  |  |  |  |  |  |  |  |  |
| **05** | 0,49 | 0,34 | 0,44 | 0,80 | 1,00 |  |  |  |  |  |  |  |  |  |  |  |  |  |  |  |  |
| **06** | 0,38 | 0,23 | 0,42 | 0,70 | 0,79 | 1,00 |  |  |  |  |  |  |  |  |  |  |  |  |  |  |  |
| **07** | 0,48 | 0,38 | 0,54 | 0,71 | 0,68 | 0,73 | 1,00 |  |  |  |  |  |  |  |  |  |  |  |  |  |  |
| **08** | 0,44 | 0,34 | 0,48 | 0,49 | 0,51 | 0,48 | 0,58 | 1,00 |  |  |  |  |  |  |  |  |  |  |  |  |  |
| **09** | 0,50 | 0,27 | 0,56 | 0,61 | 0,51 | 0,46 | 0,61 | 0,56 | 1,00 |  |  |  |  |  |  |  |  |  |  |  |  |
| **10** | 0,50 | 0,34 | 0,49 | 0,74 | 0,80 | 0,61 | 0,66 | 0,54 | 0,56 | 1,00 |  |  |  |  |  |  |  |  |  |  |  |
| **11** | 0,38 | 0,29 | 0,39 | 0,61 | 0,64 | 0,48 | 0,56 | 0,43 | 0,42 | 0,72 | 1,00 |  |  |  |  |  |  |  |  |  |  |
| **12** | 0,41 | 0,36 | 0,43 | 0,57 | 0,62 | 0,45 | 0,52 | 0,54 | 0,47 | 0,58 | 0,63 | 1,00 |  |  |  |  |  |  |  |  |  |
| **13** | 0,43 | 0,26 | 0,43 | 0,54 | 0,58 | 0,48 | 0,54 | 0,44 | 0,42 | 0,61 | 0,52 | 0,51 | 1,00 |  |  |  |  |  |  |  |  |
| **14** | 0,42 | 0,25 | 0,43 | 0,51 | 0,54 | 0,52 | 0,55 | 0,40 | 0,42 | 0,46 | 0,40 | 0,38 | 0,56 | 1,00 |  |  |  |  |  |  |  |
| **15** | 0,37 | 0,26 | 0,41 | 0,50 | 0,45 | 0,44 | 0,52 | 0,43 | 0,45 | 0,46 | 0,39 | 0,35 | 0,47 | 0,36 | 1,00 |  |  |  |  |  |  |
| **16** | 0,22 | 0,15 | 0,28 | 0,43 | 0,32 | 0,36 | 0,35 | 0,37 | 0,35 | 0,31 | 0,22 | 0,20 | 0,15 | 0,27 | 0,36 | 1,00 |  |  |  |  |  |
| **17** | 0,36 | 0,33 | 0,44 | 0,48 | 0,42 | 0,45 | 0,50 | 0,41 | 0,43 | 0,47 | 0,37 | 0,39 | 0,43 | 0,49 | 0,44 | 0,44 | 1,00 |  |  |  |  |
| **18** | 0,42 | 0,28 | 0,43 | 0,58 | 0,60 | 0,51 | 0,56 | 0,42 | 0,42 | 0,63 | 0,53 | 0,44 | 0,48 | 0,45 | 0,40 | 0,34 | 0,45 | 1,00 |  |  |  |
| **19** | 0,40 | 0,27 | 0,44 | 0,53 | 0,53 | 0,44 | 0,55 | 0,48 | 0,52 | 0,63 | 0,50 | 0,50 | 0,74 | 0,52 | 0,42 | 0,24 | 0,46 | 0,65 | 1,00 |  |  |
| **20** | 0,33 | 0,34 | 0,36 | 0,50 | 0,49 | 0,31 | 0,46 | 0,44 | 0,40 | 0,47 | 0,44 | 0,46 | 0,36 | 0,36 | 0,38 | 0,36 | 0,25 | 0,56 | 0,57 | 1,00 |  |
| **21** | 0,47 | 0,33 | 0,40 | 0,44 | 0,48 | 0,37 | 0,47 | 0,37 | 0,36 | 0,41 | 0,36 | 0,36 | 0,48 | 0,64 | 0,30 | 0,19 | 0,43 | 0,45 | 0,51 | 0,40 | 1,00 |

*Note.* *N* = 357. Items presented in the same order as in the questionnaire. All items are part of the final version of the questionnaire, as no item had to be excluded. All polychoric correlations are significant at level p < .001.

**Appendix F - Pearson-moment-correlations of EmMed Score, PA-ME Score,**

**API, API-Uro and DCS**

**Supplementary Table 8**

*Pearson-moment-correlation matrix of primary variables*

| Variable | PA-ME Score | EmMed Score | API^a^ | API-Uro^b^ | DCS^c^ |
| --- | --- | --- | --- | --- | --- |
| PA-ME Score | 1.00 |  |  |  |  |
| EmMed Score | 0.56^**^ | 1.00 |  |  |  |
| API^a^ | - 0.25 | 0.07 | 1.00 |  |  |
| API-Uro^b^ | 0.06 | 0.17 | 0.51^**^ | 1.00 |  |
| DCS^c^ | 0.22 | 0.33^*^ | - 0.16 | 0.19 | 1.00 |

*Note.* Correlations calculated within the urological sample. *n* varies due to pairwise exclusion of missing values. ^a^Participation Preference. ^b^Participation Preference regarding urological care. ^c^Decisional conflict.

^*^correlations significant at level p < .05. ^**^correlations significant at level p < .001.

**Appendix G – Results of polychoric EFAs for PAME and EmMed questionnaire in the non-clinical sample**

**PAME**

Data suitability for conducting the EFA was confirmed with the KMO test of sampling adequacy (KMO = .84, *meritorious*; 1) and a significant Barlett’s test, *χ*²(190) = 1586.62, *p* < .001. As the KMO value of two items was < .7, they were removed from analyses (2). All remaining items had a KMO value greater than .7, *range* = [0.76; 0.92]. Thus, no further item was removed.

Results of parallel analysis based on polychoric correlations suggested five factors, but in larger samples parallel analysis likely resemble over-factoring (3). Results of the Scree test, the VSS criterion, and Velicer’s MAP test allowed for either a one- or a three-factor-solution.

**EmMed**

Data suitability of the items for calculating the EFA was confirmed by a marvelous KMO measure of sampling adequacy (KMO = 0.9; *marvelous*; 1) and a significant Bartlett’s test, χ²(210) = 2184.81, *p* < .001. All items had a KMO value greater than .7, *range* = [0.85; 0.93], thus no item was removed from analyses (2).

Parallel analysis suggested a five-factor structure. However, because in larger samples parallel analysis likely resemble over-factoring (3), we decided to retain one factor in concordance with results of the Scree test, the VSS criterion, and Velicer’s MAP test. The extraction of one factor explained 39 % of variance in the EmMed items.

**References**

1. Kaiser HF, Rice J. Little Jiffy, Mark Iv. Educational and Psychological Measurement 1974; 34(1):111–7.

2. Hoelzle JB, Meyer GJ. Exploratory Factor Analysis: Basics and Beyond. In: Weiner I, editor. Handbook of Psychology, Second Edition. Wiley; 2012.

3. Warne RT, Larsen R. Evaluating a proposed modification of the Guttman rule for determining the number of factors in an exploratory factor analysis. Psychological Test and Assessment Modeling 2014; 56(1):104–23. Available from: URL: https://www.researchgate.net/profile/ross-larsen/publication/284548804_evaluating_a_proposed_modification_of_the_guttman_rule_for_determining_the_number_of_factors_in_an_exploratory_factor_analysis.
